# Microfluidic cytometric analysis of cancer cell transportability and invasiveness

**DOI:** 10.1038/srep14272

**Published:** 2015-09-25

**Authors:** Zongbin Liu, Yeonju Lee, Joon hee Jang, Ying Li, Xin Han, Kenji Yokoi, Mauro Ferrari, Ledu Zhou, Lidong Qin

**Affiliations:** 1 Department of Nanomedicine, Houston Methodist Research Institute, Houston, TX 77030, USA; 2 Department of Cell and Developmental Biology, Weill Medical College of Cornell University, New York, NY 10065, USA; 3 Department of Medicine, Weill Cornell Medical College, New York, NY 10065, USA; 4 Department of General Surgery, Xiangya Hospital, Central South University, Changsha, Hunan 410008, China; 5 Department of Molecular and Cellular Oncology, The University of Texas M. D. Anderson Cancer Center, Houston, TX 77030, USA

## Abstract

The extensive phenotypic and functional heterogeneity of cancer cells plays an important role in tumor progression and therapeutic resistance. Characterizing this heterogeneity and identifying invasive phenotype may provide possibility to improve chemotherapy treatment. By mimicking cancer cell perfusion through circulatory system in metastasis, we develop a unique microfluidic cytometry (MC) platform to separate cancer cells at high throughput, and further derive a physical parameter ‘transportability’ to characterize the ability to pass through micro-constrictions. The transportability is determined by cell stiffness and cell-surface frictional property, and can be used to probe tumor heterogeneity, discriminate more invasive phenotypes and correlate with biomarker expressions in breast cancer cells. Decreased cell stiffness and cell-surface frictional force leads to an increase in transportability and may be a feature of invasive cancer cells by promoting cell perfusion through narrow spaces in circulatory system. The MC-Chip provides a promising microfluidic platform for studying cell mechanics and transportability could be used as a novel marker for probing tumor heterogeneity and determining invasive phenotypes.

Metastasis is a set of events that occur when cancer cells break away from a primary tumor, penetrate blood or lymphatic vessels, and colonize a distant organ. Metastatic disease is often correlated with tumor progression and poor prognosis^1^,^2^. A key step in metastasis is the acquisition of increased motility and invasiveness that occurs through regulation of cell mechanical properties, such as stiffness and adhesion^3^,^4^. These mechanical properties play a critical role in cancer cell passage through narrow spaces during metastasis. Therefore, it is essential to understand how, and to what extent, mechanical properties influence cancer cell behavior. Determination of these factors could provide a label-free biomarker for cancer cells^5^. Such a marker has the potential to reduce cost and time of analyses and may provide an additional method for clinical diagnosis of cancer.

A number of biomechanical analytic methods have been utilized to probe cancer cell mechanics; these include atomic force microscopy (AFM)^6^,^7^,^8^, micropipette aspiration^9^, magnetic tweezers^10^, and optical stretching^11^,^12^. These studies consistently report that cancer cells are more flexible than normal cells and that decreased cell stiffness is correlated with increased metastatic potential. Recently, high-throughput microfluidic approaches have also been developed to characterize and enrich cancer cells based on cell mechanical properties^13^,^14^,^15^,^16^,^17^,^18^,^19^. Although significant progress has been achieved in validating cell mechanics as a label-free biomarker, current research focuses mainly on cell stiffness, or deformability, without a comprehensive consideration of size, stiffness, viscoelasticity, and cell-surface interfacial friction. It has been reported that cell-surface frictional interaction is reduced in cancer cells compared to normal cells^6^,^13^,^20^,^21^,^22^. Comprehensively measuring multiple biophysical properties and probing their combined influence on cell movement through narrow spaces may provide a more biomimetic approach for better understanding the role of cell mechanics in metastasis. Moreover, it is still difficult using current methods to carry out downstream analyses following characterization of cancer cell mechanics. Such downstream molecular analyses are particularly important for exploring the correlation between biophysical markers and molecular markers, which may offer new insight into tumor progression and initiate the discovery of new targets for diagnosis and therapy.

Here, we present a microfluidic cytometry chip (MC-Chip) that mimics cancer cell perfusion through narrow spaces of circulatory system during metastasis to study cancer cell mechanics. We utilize the microfluidic capability of particle separation and sorting for high-throughput cell-based screening of cell mechanical parameters^23^,^24^,^25^,^26^. Our MC platform possesses two key features: (1) deterministic lateral displacement (DLD), a microfluidic size-based particle-sorting technique that employs tilted rows of microposts, to separate cancer cells by size and (2) a rectangular microarray of trapping barriers with gaps decreasing in width from 15 μm to 4 μm that is comparable to blood capillary diameter ranging from 6 μm to 9 μm, to trap the cells (Fig. 1a). These features separate cells into a unique two-dimensional distribution; cells of increasing diameter are distributed across the width of the device and transportability increases in the flow direction. Cell transportability is a term that describes the effect of cell stiffness and cell-surface frictional properties, and characterizes dynamic squeezing of cancer cells through narrow spaces, as opposed to static deformation. Cancer cells, which have greater flexibility and decreased cell-surface frictional force, can be easily identified because they will be transported further within the chip.

We first demonstrated the capability of MC-Chip to achieve precise, high-throughput separation of MCF-7 breast cancer cells based on size and transportability. Next, we explored the utility of transportability to distinguish invasive basal-like breast cancer types from luminal types and demonstrate the biological significance of transportability. To analyze the impact of specific biological factors on cell transportability, we treated MCF-7 cells with the tumor promoter, 12-O-tetradecanoylphorbol-13-acetate (TPA), to increase cell invasiveness. Further, we characterized the expression of structure- and adhesion-related proteins to explore potential changes in expression profiles. The MC-Chip was further used to measure the transportability of the highly heterogeneous SUM 149 cell line, followed by downstream analysis of expression of breast cancer stem cell markers CD24 and CD44. Finally, the MC-Chip was used to study the heterogeneity of cells from a mouse tumor xenograft by comparing the transportability of cells dissociated from the center and the periphery of a tumor.

Our MC-Chip provides a biomimetic platform that may advance label-free analysis of cancer cells by high throughput, informative mechanical measurements, rapid testing, efficient data collection, and compatibility with downstream analysis. Cell transportability can potentially be used as a novel marker to probe tumor heterogeneity and indicate cancer cell invasiveness. This study presents the significance of biophysical properties in cancer cell dissemination through circulatory system.

## Results and Discussion

### Separation of cancer cells by size and transportability

**Figure 1:**
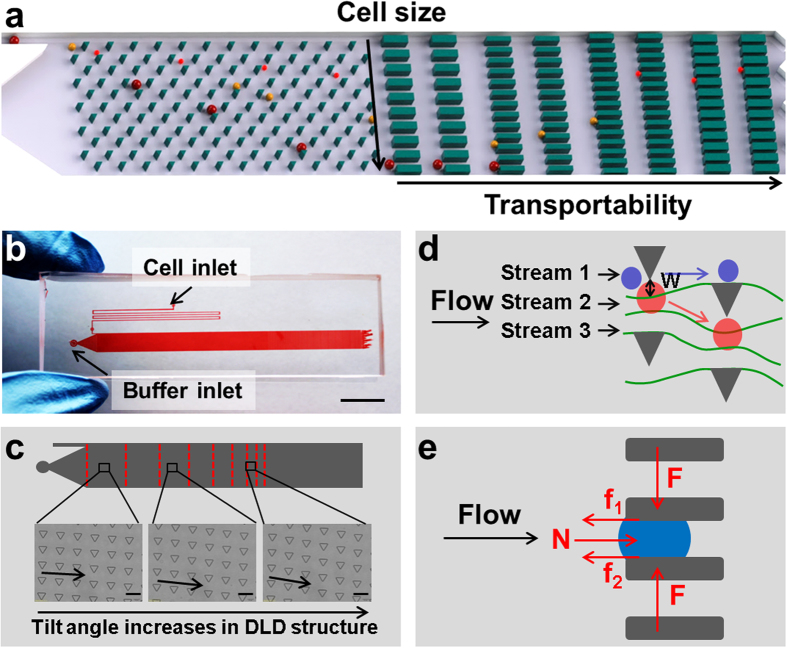
Device design and principle function. (**a**) The schematic illustrates cell separation based on size and transportability. A deterministic lateral displacement (DLD) microarray is shown on the left and a trapping barrier microarray is shown on the right. 
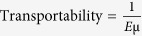
, where E is Young’s modulus and μ is friction coefficient. (**b**) The overview shows the cell and buffer inlets on the microfluidic device, scale bar = 1 cm. (**c**) DLD structure design is shown. Rows of triangular microposts with sides 30 μm in length, 27 μm in height, and separated by 30 μm gaps, are arranged with a tilt angle that gradually increases from the inlet to outlet side of the device, scale bar = 50 μm. (**d**) Cell size-based separation in the DLD structure is performed by dividing fluid flow into three streams using the microposts. Small cells (blue circles) follow the direction of fluid flow, whereas large cells (red circles) follow the direction of tilt of micropost rows, W = length of the first stream. (**e**) Stress analyses of trapped cells are performed, N = flow-induced force, arrows indicate the direction of fluid flow, F = compression force from micropost, f1 and f2 = friction forces between cell and micropost.

The layout of the MC-Chip is shown in Fig. 1b. Suspended cells within the chip flow into a micro-filter channel composed of microposts separated by 20 μm gaps to prevent them from clumping. Cells are then separated by size using the DLD structure (Supplementary Movie S1) consisting of rows of triangular microposts arranged with a tilted angle that increases from 2.9° to 14.7° between the inlet and outlet side of the device (Fig. 1c). It has been reported that triangular micropost array has better performance than circular micropost array in size-based cell separation^27^,^28^. So triangular micropost array was used in this study. Size-based separation in the DLD structure results from hydrodynamic interactions of cells with microposts under low Reynolds number conditions. As shown in Fig. 1d, fluid flow is divided into several streams by microposts. Small cancer cells, with radii smaller than the first stream (W), are able to continue following along this stream; whereas, large cells, with radii greater than W, are forced to follow the second stream along a deterministic path^29^,^30^.

After size-based separation, cancer cells are next physically captured by a matrix of trapping barriers (Supplementary Movie S2) that consists of rectangular microposts separated by gaps that decrease in width from 15 μm to 4 μm from the DLD region to the device outlet. The location of trapped cells is the result of a balance of flow-induced drag force, cell-surface frictional force, compression force from microposts, and cell elastic force (Fig. 1e). Detailed theoretical stress analysis (Supplementary Figure S1) is provided in the Supplementary Materials. From this analysis we can derive ‘transportability’, which is depicted by the following equation (Eq):


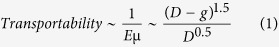


where E is ealstic modulus, μ is frcition coefficient, D is the cell diameter, and g is the gap width.

If cells are more flexible and have reduced friction coefficients, they will have high transportability and, theoretically, they will move further in the chip. In the derivation, the transportability of cells is also determined by flow rate, trapping gap width and cell diameter. To compare transportability of different cell lines, cells were perfused into chip at the flow rate. So the transportability is only determined by trapping gap width and cell diameter (Eq. 1). If cells are trapped at the same width gaps, large diameter cells have higher transportability. For a given cell, it has higher transportability if it can transport through smaller gaps.

**Figure 2:**
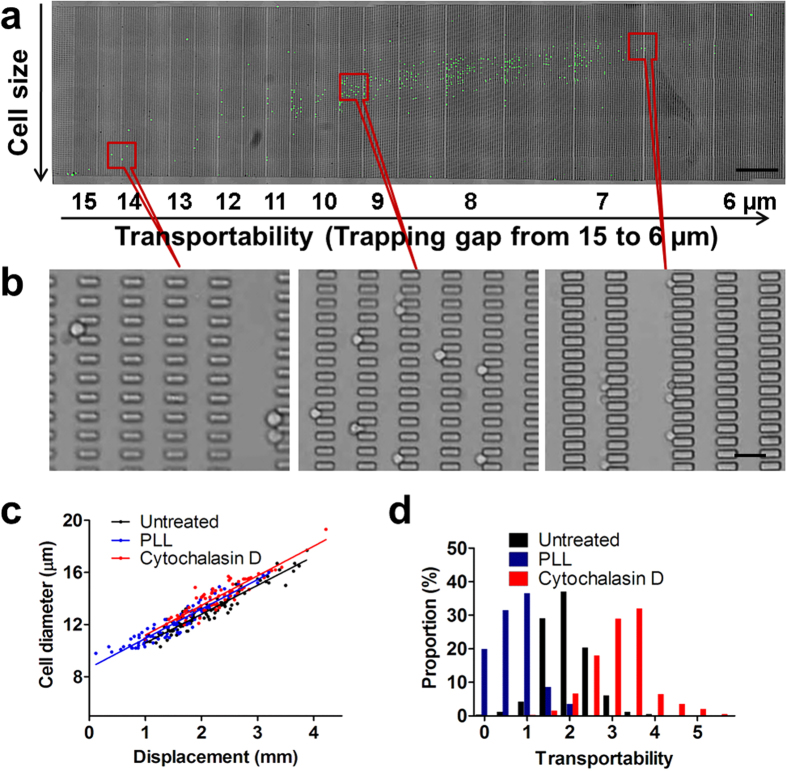
On-chip separation of MCF-7/GFP cells is achieved using the microfluidic device. (**a**) Microscopic images of fluorescence (green) and bright-field channels are merged. Cell size increases from top to bottom in the vertical direction. Transportability has horizontal distribution. The flow rate is 10 μL/min, scale bar = 1 mm. (**b**) Microscopic images show the trapped cells at indicated positions in the device, scale bar = 50 μm. (**c**) Cell diameter versus displacement is plotted for MCF-7 cells separated in untreated and PLL-treated chips, and cytochalasin D treated MCF-7 cells. (**d**) The proportion of MCF-7 cells separated in untreated and PLL-treated chips, and cytochalasin D treated MCF-7 cells with indicated transportability is shown.

To demonstrate the capability of cell separation by size and transportability, we injected a suspension of MCF-7/GFP cells into the MC-Chip. As shown in Fig. 2a,b, the separated MCF-7 cells were distributed on the chip according to two different characteristics: cell size increasing from top to bottom in the vertical direction and cell transportability increasing from left to right in the horizontal direction. A plot of cell diameter versus displacement in the vertical direction (Fig. 2c, Supplementary Figure S2 and Supplementary Figure S3) indicates a highly linear correlation between the two parameters, with a correlation coefficient (R) of 0.95. The linear relationship of cell diameter to displacement resulted from size separation in DLD structure can be described with a linear equation (*D* = 2.21*L*_*D*_+8.38, where D is the cell’s diameter and L_D_ is displacement). Given the location of a cell in the chip with the displacement and trapping gap width, cell diameter can be calculated by entering displacement into the linear equation. Alternatively, transportability can be calculated using the transportability equation (Eq. 1).

The transportability equation (Eq. 1) suggests that increasing the cell-surface frictional coefficient will reduce cell transportability within the chip. To verify this, the microfluidic channel’s surface was coated with positively charged poly-L-lysine (PLL) to increase interaction with the negatively charged cell surface. PLL treatment was associated with a slight increase in the diameter of trapped cells compared with uncoated channels at the same displacement point (Fig. 2c), and a significant reduction in cell transportability (Fig. 2d). The results of this experiment are consistent with the theoretical derivation. This experiment also suggests cell-substrate interaction has great influence on cell transportability. The MC-Chip could potentially be used to study cell-substrate interaction.

The transportability equation (Eq. 1) also suggests that decreasing cell’s elastic modulus will increase cell transportability. Cytochalasin D, which can destroy the polymerization of F-actin (Supplementary Figure S4) and reduce cell stiffness, was used to treat MCF-7 cells. Cytochalasin D treatment resulted in a slight increase of cell diameter at the same displacement. This may be due to the deformation of cells in the DLD structure, which slightly reduced cell’s effective diameter for size-based cell separation^31^,^32^. After drug treatment, the significant increasement of transportability (Fig. 2d) are also consistent with the theoretical derivation.

In the design of MC-Chip, there are total 90,000 microgaps with width ranging from 15 μm to 4 μm. One trapped cell may clog one microgap. When the quantity of trapped cells is too large, it will affect the flow rate and transportability. To study the effect of cell quantity on transportability, we compared 4,500 and 947 MCF-7 cells (Supplementary Figure S5). 4,500 cells occupied 5% of total microgaps. The transportability value of 4500 cells is 2.194, which is very close to the transportability of 947 cells (transportability value = 2.228). This transportability change can be neglected. Therefore, in all experiments of our study, the total cell number is limited to 4500.

### Transportability distinguishes breast epithelial cell lines

**Figure 3:**
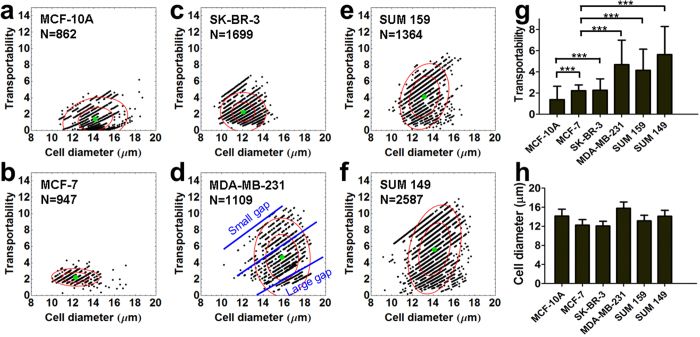
Transportabilities of six breast epithelial cell lines are compared. (**a**–**f**) Transportability versus cell diameter is plotted in MCF-10A (**a**), MCF-7 (**b**), SK-BR-3 (**c**), MDA-MB-231 (**d**), SUM 159 (**e**), and SUM 149 (**f**) breast cancer cell lines. The blue lines in (**d**) indicate the linear-like distribution of transportability and cell diameter. Top blue line shows transportability of cells trapped at smaller gap, while bottom blue line shows transportability of cells trapped at larger gap. Inner and outer red circles indicate the 50% and 90% confidence interval centered at the mean depicted by a green dot, N = number of cells counted. (**g**) The average transportability of the six breast cancer cell lines. Data are presented as mean ± s.d. ***P < 0.001. (**h**) The average cell diameter of the six breast cancer cell lines. Data are presented as mean ± s.d.

By running cell separation experiments of six well-studied breast epithelial cell lines in the MC-Chip, it was found that distinctive transportability values existed among them. The six epithelial cell lines included MCF-10A, MCF-7, SK-BR-3, MDA-MB-231, SUM 159, and SUM 149. MCF-10A are normal epithelial cells^33^. MCF-7 and SK-BR-3 are classified as breast cancer luminal types and MDA-MB-231, SUM 159, and SUM 149 are classified as breast cancer triple negative (estrogen receptor [ER]^−^, progesterone receptor [PR]^−^, and HER2^−^)/basal-like cells^34^. Triple negative/basal-like cells are often clinically more aggressive and associated with poorer prognosis than luminal cells^35^,^36^,^37^. The three basal-like cell lines show a broader transportability distribution than the normal epithelial cell line and the two luminal cell lines, which indicates increased heterogeneity in the basal-like cell types (Fig. 3a–f). Less aggressive luminal cancer cells usually contain a few dominant cell populations, whereas basal-like cancer cells have more, less-abundant genetic subpopulations^38^. In Fig. 3a–f, it is interesting that the scatter plots look like a linear distribution. Each line represents one gap width (Fig. 3d). The top line indicates cells trapped at a small gap, while the bottom line indicates cells trapped at a large gap. In each line, larger cells have higher transportability. In basal-like cell lines (Fig. 3d–f), there were more cells trapped at smaller gaps.

Average transportability was calculated for each cell line and plotted (Fig. 3g). MCF-10A has low transportability (transportability value = 1.387 ± 1.248). Luminal cell lines MCF-7 and SK-BR-3 have moderate transportability (transportability value = 2.228 ± 0.541 and 2.271 ± 1.068, respectively). The three basal cell lines MDA-MB-231, SUM 159 and SUM 149 have high transportability (transportability values = 4.682 ± 2.312, 4.149 ± 1.991, and 5.637 ± 2.637, respectively), which is 2- to 3-fold higher than the two luminal cell lines. SUM 149, which is categorized as inflammatory breast cancer^39^, the most highly metastatic variant of breast cancer characterized by rapid progression^40^,^41^, has the highest transportability. Average cell diameter was also calculated for each cell line and plotted in Fig. 3h. The average cell diameters (14.15 ± 1.45 (MCF-10A), 12.25 ± 1.16 (MCF-7), 12.07 ± 0.99 (SK-BR-3), 15.81 ± 1.30 (MDA-MB-231), 13.14 ± 1.17 (SUM 159) and 14.13 ± 1.25 (SUM 149)) show much smaller variation than the transportability (Fig. 3g). This indicates transportability of different cell lines may be more related to intrinsic mechanical properties, rather than cell size. Because transportability is determined by cell stiffness and cell-surface interaction, high transportability in the three basal-like cells indicates that there may be a reduction in one or both of these parameters, which could promote cancer cell perfusion through narrow gaps. Therefore, cancer cell transportability may be a potential biomarker for cancer cell invasiveness and metastatic potential.

### Transportability relates to cellular phenotypic shift initiated by TPA treatment

**Figure 4:**
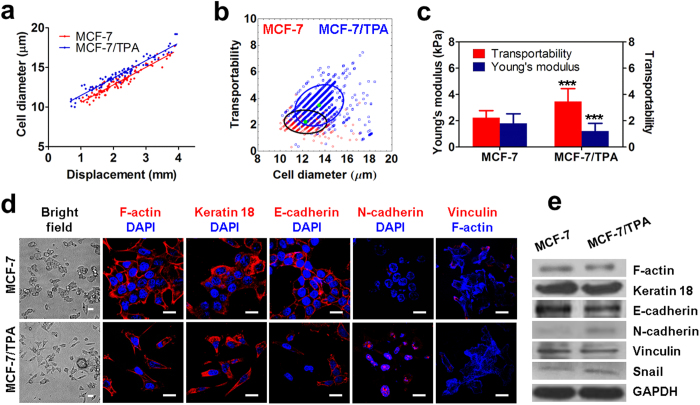
Transportability and biological parameters of untreated and TPA-induced MCF-7 cells are compared. (**a**) A plot of cell diameter distribution versus displacement shows linear correlation of these parameters for MCF-7 and TPA-induced MCF-7 cells. (**b**) Transportability versus cell diameter is plotted for MCF-7 and TPA-induced MCF-7 cells. Red and blue circles indicate the 80% confidence interval centered at the mean depicted by a green dot. The counts of MCF-7 and MCF-7/TPA are 947 and 1426 respectively. (**c**) Young’s moduli, determined by atomic force microscopy (AFM), and transportability are compared. Data are presented as mean ± s.d. ***P < 0.001 compared to MCF-7 cells. (**d**) Immunofluorescence staining of indicated proteins and counterstaining with DAPI were performed in MCF-7 and TPA-induced MCF-7 cells, scale bar = 20 μm. (**e**) Western blot analysis (cropped images) in MCF-7 and TPA-induced MCF-7 cells reveal that N-cadherin and snail are slightly up-regulated and F-actin, keratin 18, E-cadherin and vinculin are slightly down-regulated in TPA-induced MCF-7 cells. All western blot experiments were run under the same experimental conditions. Full-length blots are presented in Supplementary Figure S7.

To investigate the role of molecular and biological features in cell transportability, we treated MCF-7 cells with TPA, a potent tumor promoter, to activate protein kinase and trigger epithelial-mesenchymal transition (EMT)-like cell scattering, which describes the dispersion of epithelial cell colonies^42^. It is also reported TPA treated MCF-7 cells are softer than the untreated cells^11^. On-chip cell separation was performed to compare transportability before and after TPA treatment. Western blotting and immunofluorescence analyses were carried out to characterize changes in biological parameters of cells. On-chip separation showed that untreated and TPA-induced MCF-7 cells show a linear correlation between cell diameter and displacement, with a correlation coefficient R of 0.95 for MCF-7 and 0.94 for MCF-7/TPA cells (Fig. 4a). At the same displacement, there is a shift of cell diameter toward greater value. Similar to cytochalasin D drug treatment, TPA treatment decreased cell stiffness (Supplementary Figure S6). In the DLD structure, shear stress forced slight cell deformation and decreased cell’s effective diameter, inducing the shift of cell diameter toward greater value at the same displacement^31^,^32^. Figure 4b shows the relationship between cell diameter and transportability in MCF-7 and MCF-7/TPA cells. The histogram indicates that there are significant shifts toward greater cell diameter and transportability after TPA treatment. We further measured stiffness of MCF-7 and MCF-7/TPA cells using AFM to correlate transportability with cell stiffness (Supplementary Figure S6). After TPA treatment, the average Young’s modulus decreases from 1.80 ± 0.73 kPa to 1.21 ± 0.59 kPa, whereas transportability increases from 2.228 ± 0.541 to 3.476 ± 0.976 (Fig. 4c). This suggests that more flexible cells can more easily pass through small gaps.

In addition to characterizing cellular mechanics by MC-Chip, we analyzed the expression of a variety of cell structure- and adhesion-related proteins, including F-actin, keratin 18, vimentin, E-cadherin, N-cadherin, vinculin and also one EMT marker snail. As shown in the bright field images (Fig. 4d), MCF-7 cells are closely attached, unlike MCF-7/TPA, which appear as elongated single cells. The immunofluorescent staining (Fig. 4d) and western blotting (Fig. 4e and supplementary Figure S7) results show that there is slight down-regulation of F-actin, E-cadherin, vinculin and slight up-regulation of N-cadherin and snail after TPA treatment. There is no expression of vimentin in both untreated and TPA treated MCF-7 cells (data is not shown). Down-regulation of F-actin and vinculin may contribute to the decrease of cell stiffness and cell-surface interaction, and thus, increase cell transportability. Based on the results, TPA treatment may induce more invasive phenotypes in MCF-7 cells, which may lead to increased cell transportability within the chip.

### Correlation of high transportability with up-regulated cancer stem cell marker expression

**Figure 5:**
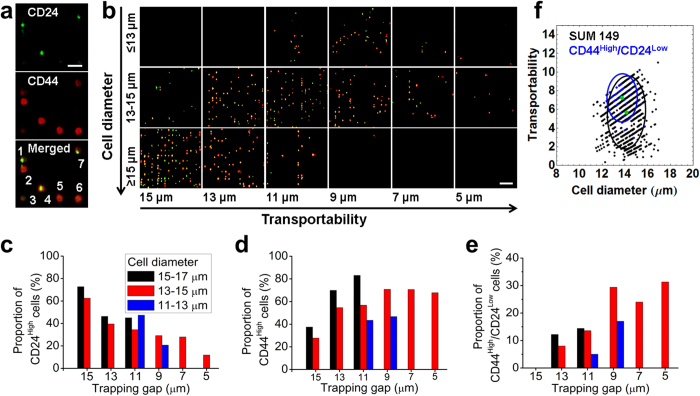
The relationship between CD24/CD44 expression and transportability in SUM149 cells is investigated. (**a**) Immunofluorescence staining is shown for CD24 (green), CD44 (red), and the merged image. The cells labeled 1 and 7 are CD24^High^/CD44^Low^ phenotypes. The cells labeled 2, 5, and 6 are CD24^Low^/CD44^High^ phenotypes. The cell labeled 4 is CD24^High^/CD44^High^ phenotype. The cell labeled 3 is CD24^Low^/CD44^Low^ phenotype, scale bar = 50 μm. (**b**) Microscopic images show CD24 and CD44 staining of trapped cells of indicated diameters and transportability, scale bar = 100 μm. (**c**) The proportion of CD24^High^ cells is plotted versus gap width. The proportion of cells expressing CD24 decreases with transportability. (**d**) The proportion of CD44^High^ cells is plotted versus gap width. The proportion of cells expressing CD44 increases with transportability. (**e**) The proportion of CD24^Low^/CD44^High^ cells is plotted versus gap width. The proportion of cells with phenotype CD24^Low^/CD44^High^ increases with transportability. (**f**) Transportability versus cell diameter is plotted for CD24^Low^/CD44^High^ cells and SUM 149. Blue and black circles indicate the 80% confidence interval centered at the mean depicted by a green dot.

Cancer cells are genetically and functionally heterogeneous across different types of cancer and within subtypes of the same cancer. Techniques such as fluorescence-activated cell sorting have been used to sort cancer cells based on molecular phenotype. The MC-Chip we describe here can be used to explore cancer cell heterogeneity based on biophysical parameters. To probe cancer heterogeneity and identify associations between biophysical and biochemical parameters, we used the MC-Chip to separate cells from a highly heterogeneous breast cancer line, SUM 149, which has been reported to contain a minor subpopulation of cancer stem cells^43^. After cell separation, we performed on-chip staining for breast cancer stem cell-related biomarkers CD24 and CD44 (Fig. 5a). As shown in Fig. 5b–d, CD24 expression is down-regulated and CD44 expression is up-regulated, with increased cell transportability. Moreover, cells with high transportability had a greater number of cells with the CD24^Low^/CD44^High^ phenotype (Fig. 5e). The transportability of CD24^Low^/CD44^High^ phenotype cells in whole SUM 149 population is shown in Fig. 5f. The average transportability of CD24^low^/CD44^high^ cells is 7.238, higher than the transportability of SUM 149 (transportability value = 5.67).

Recent work reported that breast cancer cells with the CD24^Low^/CD44^High^ phenotype are enriched in tumor-initiating cancer stem cells and are highly invasive^44^,^45^,^46^. Correlation of high transportability and enrichment of CD24^Low^/CD44^High^ cells may suggest that cell transportability can be used as a potent biomarker of cancer cell stemness. In this study, the MC-Chip with trapped cancer cells can be used as a novel and disaggregated biopsy that contains information on cell mechanics and is compatible with downstream immunofluorescence analyses. Though immunofluorescence analysis may correlate the cell mechanics with protein biomarker expression, it is more meaningful to do further analysis such as gene expression. To do this analysis, it’s necessary to sort cells based on cell mechanics. However, the MC-Chip cannot achieve this function, which is currently the limitation of our chip.

### The tumor periphery contains more high-transportability tumor cells than the tumor center

**Figure 6:**
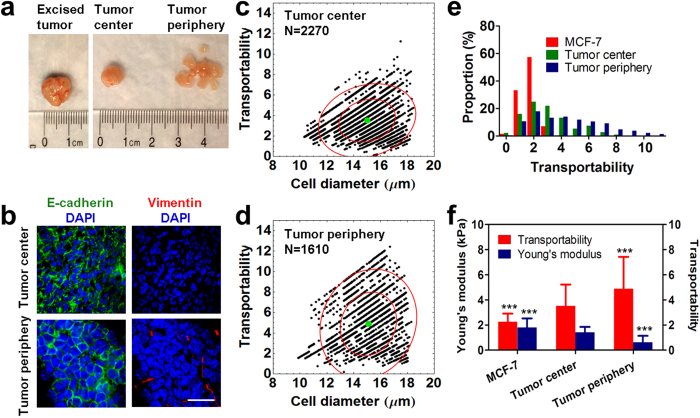
Transportability and biological parameters of tumor cells dissociated from the tumor center and periphery are compared. (**a**) Images show the excised tumor, tumor center, and tumor periphery. (**b**) Fluorescence images show E-cadherin (green) and vimentin (red) staining of cells from the tumor center and periphery. Cells were counterstained with DAPI. E-cadherin is down-regulated and vimentin is up-regulated in cells from tumor periphery, scale bar = 20 μm. (**c**,**d**) Transportability is plotted versus cell diameter for cells from the tumor center (**c**) and tumor periphery (**d**). Inner and outer red circles indicate the 50% and 90% confidence interval centered at the mean depicted by a green dot, N = number of cells counted. (**e**) The proportion of indicated cell types with indicated levels of transportability is given. The tumor periphery contains more cells with high transportability. (**f**) A comparison of Young’s moduli and transportability is made in indicated cells. Cells from the tumor periphery are more flexible and have higher transportability than MCF-7 cells and cells from the tumor center. Data are presented as mean ± s.d. ***P < 0.001 compared to tumor center cells.

It has been reported that tumor cells are highly heterogeneous in both primary and metastatic tumors. In the central part of a tumor, cells express higher levels of epithelial markers. In contrast, cells from the tumor periphery have undergone EMT and express higher levels of mesenchymal markers that facilitate tumor cell invasion into adjacent tissues^47^,^48^,^49^. To probe the heterogeneity of primary tumor cells and further explore the predictive potential of transportability for invasive phenotypes, we used the MC-Chip to separate cells dissociated from the center and periphery of a mouse tumor xenograft of MCF-7 cells (Fig. 6a). On-chip staining with an anti-human nucleus antibody was used to identify cancer cells^50^. Supplementary Figure S8 and Supplementary Table S1 show that most cells trapped in chip are tumor cells and not host-derived cells. Tumor cell transportability was calculated and as shown in Fig. 6c–e, the average transportability of tumor center cells is 3.508 ± 1.718, which is higher than that of MCF-7 cells (2.228 ± 0.541). The tumor periphery contains more cells with higher transportability, and has the highest average transportability (4.892 ± 2.536). Transportability values were then compared to Young’s moduli derived from AFM measurements (Fig. 6f). Decreased Young’s modulus resulted in a conspicuous increase in transportability.

To demonstrate the biological differences between cells from the tumor center and periphery, mouse tumors were further analyzed by immunohistochemical staining of E-cadherin and vimentin (Supplementary Figure S9 & S10). In the central region of the tumor, there is an abundance of cell-cell junctions with E-cadherin expression (Fig. 6b and Supplementary Figure S10); however, vimentin is not expressed. Alternatively, E-cadherin expression is down-regulated and vimentin expression is up-regulated in the tumor periphery. Loss of E-cadherin expression and a gain in vimentin expression are associated with EMT and increased invasiveness. Taken together, the transportability and immunohistochemical results demonstrate that the tumor periphery is enriched with a greater number of invasive tumor cells with higher transportability than the tumor center. This experiment, using animal tumor samples, demonstrates the capability of the MC-Chip to identify invasive cell phenotypes from heterogeneous populations of biological samples.

## Conclusions

Here, we describe a biomimetic MC-Chip that precisely separates large populations of cells based on cell diameter and transportability. Cell transportability describes the overall effects of cell stiffness and cell-surface frictional properties. Decreased cell stiffness and cell-surface interactions may promote cancer cell perfusion through narrow spaces during metastasis. MC-Chip analysis of a panel of breast cancer cell lines reveals that more invasive basal-like cell types have higher transportability and greater heterogeneity than the less invasive luminal cell types. Analysis of TPA-induced cancer cells demonstrates that TPA treatment may alter expression of cell structure- and adhesion-related proteins, which ultimately increases cell transportability. We also used the MC-Chip to explore the relationship between transportability and cancer stem cell marker expression in the heterogeneous cell line SUM 149. Our results suggest that in these cells, CD24 expression is down-regulated and CD44 expression is up-regulated with an increase in transportability. The platform was further applied to analyze cells from a mouse tumor xenograft, and we found that compared to cells from the tumor center, cells from the tumor periphery have higher expression of invasive molecular features and higher transportability. In summary, we have successfully developed a biomimetic platform to separate cells based on size and mechanical-phenotype. We demonstrate that our microfluidic platform can probe the heterogeneity of cancer cell mechanics, identify invasive phenotypes, and reveal the association between cell’s biophysical properties and biomarker expression.

## Methods

### Device fabrication and operation

The microfluidic device was fabricated according to standard photolithography and soft lithography procedures. The negative photoresist SU8-3025 (MicroChem, Newton, MA) pattern on a silicon wafer was fabricated with a photomask. The silicon wafer was then silanized using trimethylchlorosilane (TMCS) (Thermo Scientific, Waltham, MA) to facilitate polydimethylsiloxane (PDMS) mold release. PDMS prepolymer (Sylgard 184 silicone elastomer kit; Dow Corning, Midland, MI) was poured onto the silicon wafer and cured at 80 °C for 1 h. Holes were punched in the PDMS, and oxygen plasma treatment was used to chemically bond the PDMS mold to a glass slide.

The fabricated microfluidic device has two inlets (one for cells and the other for buffer) and one long channel between the inlets and outlet (Fig. 1b). The channel dimensions are 60 mm long, 4.4 mm wide, and 30 μm high. The cell inlet is connected to a microfilter channel designed with microposts 20 μm in diameter and separated by 20 μm gaps. The DLD structure, connected to the buffer inlet, consists of rows of triangular microposts with sides 30 μm in length and separated by 30-μm gaps. The rows are arranged with a tilt angle that increases from 2.9° to 14.7° between the inlet and outlet sides of the device. The trapping barrier structure connected to the DLD microarray is composed of rectangular microposts with sides 25 μm × 10 μm in length, separated by gaps decreasing in width from 15 μm to 4 μm between the inlet side and the outlet end of the device.

To separate cancer cells, the microfluidic device was first blocked with 1% bovine serum albumin (BSA) in phosphate buffered saline (PBS) for 1 h and then washed with cell culture medium. For the PLL coating experiment, PLL solution (Sigma, St. Louis, MO) was incubated in the chip for 1 h. Cells suspended in the solution at 5 × 10^6^ cells/mL were injected into the device at a flow rate of 0.5 μL/min. The flow rate of the medium was maintained at 10 μL/min using a syringe pump. Next, cells were physically captured on the chip. Computational fluid dynamics (CFD) simulations and theoretical stress analyses were used to analyze the captured cells. From this analysis, transportability was derived. See Supplementary Materials for detailed methods concerning CFD simulation, stress analyses, and derivation of transportability.

Trapped cancer cells were restored to their original round shape when flow stress was released (Supplementary Figure S3). The restoration lasted less than 4 min. Then Olypus cellSens Dimension software was used to analyze cell image and cell diameter was obtained as the mean of long axis and short axis. A linear equation can be derived from the linear fit between cell diameter and displacement, which is resulted from size separation in DLD structure. Given the location of each cell in the chip, information on displacement and width of the trapping gap were obtained from the software. Cell diameter was calculated by entering displacement into the linear equation. Transportability was calculated by entering values for cell diameter and gap width into the transportability equation. Mathematics software was used to generate the plots of transportability versus cell diameter. The proportion of cells as a function of cell diameter or transportability was determined.

### Cell culture and preparation

The cell lines MCF-7 and MDA-MB–231 (ATCC, Manassas, VA) were cultured in Dulbecco’s Modified Eagle Medium (DMEM; Corning, Manassas, VA) supplemented with 10% (vol/vol) fetal bovine serum (FBS) and 1% penicillin-streptomycin. For TPA treatment, MCF-7 cells were cultured in the same medium supplemented with an additional 100 nM TPA (Sigma, St. Louis, MO) for 7 days. The MCF-10A cell line was cultured in EMEM/F12 medium (Lonza, Walkersville, MD) supplemented with 5% (vol/vol) horse serum, 20 ng/mL EGF, 0.5 mg/mL hydrocortisone, 100 ng/mL cholera toxin, 10 μg/mL insulin and 1% penicillin-streptomycin. The SK-BR-3 cell line (ATCC) was cultured in McCoy’s 5A medium (HyClone, Logan, Utah) supplemented with 10% (vol/vol) FBS and 1% penicillin-streptomycin. SUM 149 and SUM 159 cell lines (Asterand, Detroit, MI) were cultured in Ham’s F-12 medium (Lonza) supplemented with 5% (vol/vol) FBS, 5 μg/mL insulin, 1 μg/mL hydrocortisone, and 1% penicillin-streptomycin. Cell cultures were maintained at 37 °C in a humidified atmosphere of 5% (v/v) CO_2_. To synchronize cells, cells were cultured in serum-free medium for 24 h. After synchronization, cells were harvested by incubation in trypsin for 5 min followed by centrifugation at 188 × *g* for 3 min. The cell suspension was then diluted to the desired concentration with serum-free medium.

### Western blotting analysis

MCF-7/GFP and TPA-induced MCF-7/GFP cells were lysed with Pierce® RIPA buffer (Thermo Scientific) containing a protease inhibitor cocktail (Thermo Scientific). Cell lysates (10 μg protein) in loading buffer (LDS sample buffer, non-reducing; Thermo Scientific) were heated for 5 min at 95 °C. Cell lysates and protein ladder (Xpert 2 Prestained Protein Marker, GenDEPOT, Barker, TX) were loaded onto polyacrylamide gels (Any kD^™^ Mini- PROTEAN^®^ TGX^™^ Precast Gel, Bio-Rad, Hercules, CA) and transferred to nitrocellulose membranes (Bio-Rad). The blots were blocked in TBST (20 mM TRIS, pH 7.6, 150 mM NaCl, 0.05% Tween 20) containing 5% milk for 1 h at room temperature. The blots were incubated overnight in TBST/5% milk containing primary antibodies anti-E-cadherin (1:1000; Abcam, Cambridge, UK), anti-N-cadherin (1:500, Cell Signaling Technology, Danvers, MA), anti-vinculin (1:1000, Abcam), anti-keratin 18 (Cell Signaling Technology), anti-F-actin (1:1000, Abcam), anti-vimentin (1:1000, Abcam), anti-snail (1:1000, Abcam) and anti-GAPDH (1:2000, Cell Signaling Technology). Blots were washed three times with TBST and incubated in TBST/5% milk containing the secondary antibodies anti-mouse IgG conjugated to horseradish peroxidase (HRP) (1:5000, Cell Signaling Technology) or anti-rabbit IgG conjugated to HRP (1:5000, Cell Signaling Technology) for 1 h at room temperature. Blots were then placed on a mixture of Pierce® ECL western blotting substrate (Thermo Scientific) and Super Signal West Femto Maximum Sensitivity Substrate (Thermo Scientific). Proteins were detected by chemiluminescence.

### Immunofluorescence imaging

MCF-7 and MCF-7/TPA cells were seeded in 6-well plates on sterile Corning® glass coverslips (22 mm × 22 mm) at a density of 2 × 10^5^ cells/well and incubated for 24 h. The cells were fixed with 4% paraformaldehyde (Affymetrix, Santa Clara, CA) for 30 min at room temperature and then permeabilized with PBS containing 2% Triton X-100 (Sigma-Aldrich, St. Louis, MO) and blocked with 2% BSA (Calbiochem, San Diego, CA) for 1 h. Cells were incubated with anti-E-cadherin antibody (1:100, Abcam), anti-N-cadherin antibody (1:50, Abcam), anti-vinculin antibody (1:200, Abcam), anti-F-actin (1:100, Abcam), anti-vimentin (1:100, Abcam), anti-keratin 18 antibody (1:100, Cell Signaling Technology), or anti-lamin A/C antibody (1:200, Cell Signaling Technology) overnight at 4 °C with gentle shaking. Cells were washed twice with PBS for 10 min and incubated with Alexa Fluor 594-conjugated secondary antibodies for 1 h at room temperature. Nuclei were stained with Molecular Probes® Hoechst 33342 (Life Technologies, Grand Island, NY) for 10 min. Cells probed with the anti-vinculin antibody were further incubated with DyLight 350-conjugated phalloidin (1:250, Thermo Scientific) for 1 h at room temperature to simultaneously visualize F-actin and vinculin. To stain only F-actin, cells were permeabilized with PBS containing 2% Triton X-100 and blocked with 2% BSA for 1 h and then incubated with Alexa Fluor® 594-conjugated phalloidin (1:250; Life Technologies) for 1 h at room temperature. The stained cells were monitored using a confocal laser-scanning microscope (Olympus FluoView FV1000).

### Tumor cell isolation from mouse tumor xenograft

Orthotopic models from MCF-7 cell lines were established for these studies. Tumor models were established in Athymic nude mice (Charles River Laboratories, Wilmington, MA) with a one-time injection of tumor cells/Matrigel 1:1 mixture into the mammary fat pad region. A volume of 100 μL of the cell suspension (1 × 10^7^ cells/mL) was injected. When the volume of the tumor reached approximately 1000 mm^3^, tumor-bearing mice were sacrificed, and the tumors were removed and cut into center and periphery parts. The resulting parts were then cut into small pieces using scalpels in sterile PBS. The tumor tissue was digested with collagenase type 3 (2 mg/mL, Worthington Biochemical, Lakewood, NJ) for 1 h at 37 °C. The harvested cell suspension was filtered through a 40-μm nylon cell strainer (BD Biosciences), centrifuged at 200 × *g* for 5 min and then re-suspended in PBS. After centrifugation, cells were suspended in Dulbecco’s Modified Eagle Medium (DMEM) supplemented with 10% FBS. The resulted tumor cells were separated by MC-Chip following the procedure described above.

#### Ethical statement

All animal experiment procedures were approved by the Institutional Animal Care and Use Committee of Houston Methodist Research Institute according to the animal standards of care of the National Institutes of Health.

### Histochemical staining

Tumor tissues were embedded in Tissue-Tek® O.C.T. Compound (Fisher Scientific) and were cryosectioned at 5-μm thickness. Sections were subsequently stained with Hematoxylin and eosin (H&E stain) and examined with bright light microscopy. The slices were also examined by laser-scanning confocal microscopy (Olympus FluoView FV1000). After fixing tissue slices with 4% paraformaldehyde for 20 min at room temperature, they were blocked and permeabilized with 3% BSA in PBS with 0.2% Triton X-100 for 20 min at room temperature. Tissue slices were incubated for 1 h with anti-E-cadherin and anti-vimentin (Abcam) at room temperature with gentle shaking. They were washed twice with PBS for 10 min and incubated with Alexa 488- (for E-cadherin) 594- (for vimentin) conjugated secondary antibodies for an hour at room temperature. After washing with PBS, the nuclei were stained using Hoechst 33342 (Life Technologies) for 10 min. Tissue slices were analyzed.

### Statistical analysis

All data are presented as mean ± s.d. The non-parametric test (Wilcoxon-Mann-Whitney test) was used to compare two groups in GraphPad software (*P* < 0.05 was considered significant).

## Additional Information

**How to cite this article**: Liu, Z. *et al.* Microfluidic cytometric analysis of cancer cell transportability and invasiveness. *Sci. Rep.* **5**, 14272; doi: 10.1038/srep14272 (2015).

## Supplementary Material

Supplementary Information

Supplementary Movie S1

Supplementary Movie S2
