# Supplementary material for: Microfluidic cytometric analysis of cancer cell transportability and invasiveness
Source: Sci Rep. 2015 Sep 25;5:14272. doi: 10.1038/srep14272 (PMC4585905; doi:10.1038/srep14272)
Supplement: Supplementary Information [file srep14272-s1.pdf]

Supplementary Information

Microfluidic cytometric analysis of cancer cell transportability and invasiveness

Zongbin Liu[1,2], Yeonju Lee[1], Joon hee Jang[1,2], Ying Li[1,2], Xin Han[1,2], Kenji Yokoi[1], Mauro Ferrari[1,3], Ledu Zhou[1,2,4,*], Lidong Qin[1,2,5,*]

[1]Department of Nanomedicine, Houston Methodist Research Institute, Houston, TX 77030, USA
[2]Department of Cell and Developmental Biology, Weill Medical College of Cornell University, New York, NY 10065, USA
[3]Department of Medicine, Weill Cornell Medical College, New York, NY 10065, USA
[4]Department of General Surgery, Xiangya Hospital, Central South University, Changsha, Hunan 410008, China
[5]Department of Molecular and Cellular Oncology, The University of Texas M. D. Anderson Cancer Center, Houston, TX 77030, USA

*Correspondence and requests for materials should be addressed to L.Q. (LQin@houstonmethodist.org) or L.Z. (LZhou@houstonmethodist.org).

Methods

Simulation and modeling. The velocity distribution of fluid flow in the trapping barrier structure was analyzed based on the laminar flow law of Newton fluid using commercial software for computational fluid dynamics (CFD) simulation[1,2]. GAMBIT software was used to establish a 3D microfluidic system. Then, the mesh file was introduced into ANSYS FLUID software by setting proper compute parameters under acceptable grid densities and local grid refining. In the CFD simulation, the laminar flow module was selected, which enabled the motion type present in the system to be determined and the flow velocity profile to be analyzed by the Navier-Stokes equations for an isothermal incompressible fluid. The viscosity and density of the buffer solution were assumed to be identical to that of water.

AFM measurements. MCF-7 and TPA-induced MCF-7 cells were cultured on 6-cm cell culture dishes and all measurements were performed in culture medium at 37°C. The AFM (Bioscope, Bruker Corporation, Billerica, MA) was equipped with an inverted light microscope (Olympus IX81) so that the conditions of the live cells were constantly monitored. Silica microparticle (diameter of 5 µm) modified silicon nitride cantilevers (Novascan Technologies, Ames, IA) with approximate spring constant values of ~ 0.06 N/m were employed to measure cell stiffness. The exact spring constant value was measured by thermal tuning method. Probes were positioned at the cells' nuclei proximities under optical control, and force curves were acquired at a sample rate of 1Hz with an indentation force of 10 pN. The calculation of Young's modulus was performed based on the Hertz model using the Nanoscope analysis program from Bruker corporation, where F = force, E = Young's modulus, ν = Poisson's ratio (ν = 0.5, in this study), R = radius of the indenter (R = 2500 nm, in this study), and δ = indentation depth. To obtain Young's moduli, at least 80 cells were measured.

$$F = \frac{4}{3}\frac{E}{(1-\nu^2)}\sqrt{R}\delta^{3/2}$$

After measurement of single cell force spectroscopy, cells were fixed with 4% paraformaldehyde for 30 min at room temperature and washed twice with PBS. AFM imaging of fixed cells was carried out in PBS at room temperature. The AFM was operated in the contact mode using a MLCT-C cantilever (nominal spring constant, k = 0.01 N/m, Bruker Corporation, Billerica, MA) at a scan rate of 1 Hz. The AFM images were processed using the Nanoscope analysis program.

On-chip immunofluorescence imaging for tumor cell identification. Tumor cells, dissociated from the mouse tumor center and periphery, were flowed into the microfluidic chip that had been pre-coated with 1% BSA-containing PBS buffer. The trapped cells were fixed and permeabilized following the same protocol described above. Next, the cells were incubated with the anti-nucleus antibody (1:100, Millipore, Billerica, MA) in 3% BSA-containing PBS solution. After rinsing 2-3 times with PBS, cells were incubated with Alexa 488-conjugated secondary antibody for 1 h. Finally, cell nuclei was counterstained using Hoechst 33342 (Life Technologies) for 10 min, followed by PBS rinsing for 2-3 times. The stained cells were observed with an Olympus IX81 inverted fluorescence microscope.

Results

Simulation and stress analysis. Supplementary Figure S1a shows the simulated flow rate distribution in the microfluidic chip trapped with cancer cells. The simulation suggested there was uniform distribution of flow rate in the interval between the two rows of microposts. The flow rate near the surface of the cells was reduced to zero. Supplementary Figure S1b shows an

image of trapped cells. To facilitate the stress analysis, cells were regarded as elastic spheres. The forces applied to cells included flow induced force, cell-post surface maximum static friction force, and compression force from the micropost (Supplementary Figure S1c). Since the cell diameter (10-20 μm) was smaller than the height of the micropost (27μm) and cells had equivalent gravity and buoyancy in the vertical direction, the trapped cells were assumed to be suspended in the gap without contacting the upper surface, and the friction force between cells and lower surface was neglected compared to that between cells and microposts. Based on the force balance, the equation,

$N = f1 + f2$ Eq 1

can be derived, where N ($kg \cdot m \cdot s^{-2}$) is the drag force induced by flow and f1 and f2 ($kg \cdot m \cdot s^{-2}$) are friction forces between the cell and micropost. In this study, the flow in the microchannel is laminar with extremely low Re (Re<<1). Drag force can be calculated based on the Stoke's law. However, the Stoke's law describes the drag force on spherical objects. The trapped cells have non-spherical shape and the shape factor[3] is considered to calculate the drag force by

$N = 3\pi\sigma vDK$ Eq 2

where σ ($kg \cdot s^{-1} \cdot m^{-1}$) is the dynamic viscosity, v ($m \cdot s^{-1}$) is the initial flow rate, D (m) is the cell diameter, and K is the shape factor, which is determined by the shape of an object[3]. Since the elastic modulus of cells is far lower than that of PDMS[4,5], the force F can be calculated based on the Hertzian contact model[6]

$F = \frac{Ea^3}{3D}$ Eq 3

where E ($m \cdot kg \cdot s^{-2}$) is the elastic modulus of the cell, a (m) is the diameter of the circular contacting area between the cell and micropost, and D (m) is the cell diameter. Based on the cell

deformation in the confined channel (Supplementary Figure S1d)[6], the contact length can be described by

$$a \sim \sqrt{2D(D-g)} \qquad \text{Eq 4}$$

where g (m) is the gap width. From Eq 3 and 4, F is then calculated

$$F = 0.471ED^{0.5}(D-g)^{1.5} \qquad \text{Eq 5}$$

From Eq 5, friction is calculated by

$$f1 = f2 = F\mu = 0.471E\mu D^{0.5}(D-g)^{1.5} \qquad \text{Eq 6}$$

where μ is the friction coefficient. Finally from Eq 1, 2, and 6, we get

$$\frac{1}{E\mu} \sim \frac{0.207(D-g)^{1.5}}{\pi\sigma v K D^{0.5}} \qquad \text{Eq 7}$$

where E is the elastic modulus, μ is the friction coefficient, σ is the dynamic viscosity which is a constant, v is the flow rate, g is the gap width, D is the cell diameter, and K is the shape factor. From the simulation results, v can be regarded as a constant. K is determined by cell shape. Cells have similar shape between microposts when they squeeze through gaps. The trapped cell can be simplified to a cylinder, indicated by the blue line in Supplementary Figure S1c. In the study of David leith, K is around 1.5 for a cylinder[3]. Therefore, we approximate that K remains nearly constant for each cell. Then, Eq 7 can also be written as

$$\frac{1}{E\mu} \sim \frac{(D-g)^{1.5}}{D^{0.5}} \qquad \text{Eq 8}$$

$\frac{1}{E\mu}$ is depicted as transportability. If cells are more flexible and have reduced friction coefficients, they will have high transportability and, theoretically, they will move further in the chip. From Eq 7, the transportability of cells is also determined by flow rate, trapping gap width and cell diameter. To compare transportability of different cell lines, cells were perfused into chip at the flow rate. So transportability is only determined by trapping gap width and cell diameter (Eq 8).

If cells are trapped at the same width gaps, large diameter cells have increased transportability. For a given cell, it will have higher transportability if it can transport through smaller gaps.

In the above derivation of transportability, we consider cells as elastic spheres. However, cells are actually viscoelastic and possess both elastic and viscous properties. When cells perfuse from the initial gap to the next smaller gap, there is not enough time for stress relaxation due to their viscous properties. The remained deformation before entering the next gap may facilitate subsequent deformation and makes cells easily transport through gaps. Thus, the viscous properties of cells may increase cell's transportability.

Effect of flow rate on cell size separation in the DLD structure. To study the influence of flow rate on cell size separation, we compared the effect of two flow rates, 10 μL/min and 15 μL/min. As shown in Supplementary Figure S2, increased flow rate has no impact on linear correlation of cell diameter and displacement. At the same displacement, a flow rate of 15 μL/min results in slightly increased diameters of trapped cells compared to the flow rate of 10 μL/min. It was also found that all the cells were trapped in the chip at 10 μL/min, while only part of the cells were trapped at 15 μL/min. Thus the flow rate of 10 μL/min was used in the experiment.

AFM measurement of MCF-7 and MCF-7/TPA cells. After TPA treatment, the morphology of MCF-7 cells changed from epithelial-like to mesenchymal-like (Supplementary Figure S6). The Young's moduli collected from AFM measurements also showed a significant difference between MCF-7 and MCF-7/TPA. The MCF-7/TPA cells had reduced Young's moduli (Supplementary Figure S6b). Decreased cell stiffness benefits cell deformation when squeezing through small gaps, which may explain why MCF-7/TPA had increased transportability in chip.

Tumor cell identification. Tumor cells were identified by human specific anti-nucleus staining. Supplementary Figure S8 shows the merged microscopic images of on-chip staining in bright-field: green for anti-nucleus and blue for DAPI. Most cells are both anti-nucleus and DAPI positive, indicating that the trapped cells are tumor cells, rather than host derived cells. The proportion of tumor cells in total cells was calculated (Supplementary Table S1). The tumor center had 97.1% cancer cells, whereas the tumor periphery had 95.1% cancers cells.

Immunohistochemical staining. Mouse tumors were further analysed by H&E (Supplementary Figure S9) and immunohistochemical staining of E-cadherin and vimentin (Supplementary Figure S10). Tumor cells from the center had high expression of E-cadherin and no expression of vimentin. In contrast, tumor cells from the periphery had down-regulated expression of E-cadherin and up-regulated expression of vimentin.

1 Li, Y., Xu, Y., Feng, X. & Liu, B. F. A rapid microfluidic mixer for high-viscosity fluids to track ultrafast early folding kinetics of G-quadruplex under molecular crowding conditions. *Anal. Chem.* **84**, 9025-9032 (2012).

2 Mengeaud, V., Josserand, J. & Girault, H. H. Mixing processes in a zigzag microchannel: finite element simulations and optical study. *Anal. Chem.* **74**, 4279-4286 (2002).

3 Leith, D. Drag on Nonspherical Objects. *Aerosol. Sci. Tech.* **6**, 153-161 (1987).

4 Kuznetsova, T. G., Starodubtseva, M. N., Yegorenkov, N. I., Chizhik, S. A. & Zhdanov, R. I. Atomic force microscopy probing of cell elasticity. *Micron.* **38**, 824-833 (2007).

5 Palchesko, R. N., Zhang, L., Sun, Y. & Feinberg, A. W. Development of polydimethylsiloxane substrates with tunable elastic modulus to study cell mechanobiology in muscle and nerve. *Plos One* **7**, e51499 (2012).

6 Popove, V. L. Contact mechanics and friction. (2010).

Figure S1 | Simulation and stress analysis. (a) Computational modeling shows flow rate distribution in the microfluidic device. The flow rate has uniform distribution across the channel. (b) The image shows a cell trapped in the gap, scale bar = 10 μm. (c) The image shows top, front, and side views and stress analysis of a cell trapped in a gap, N = flow-induced force, F = compression force from post, f1 and f2 = friction forces between cell and micropost, g = gap width, a = diameter of the circular contacting area between the cell and micropost. (d) The schematic diagram shows cell deformation in the gap, g = gap width, a = diameter of the circular contacting area between the cell and micropost.

Figure S2 | Flow rate affects size-based cell separation. Higher flow rate is associated with larger cells than lower flow rate at the same degree of displacement.

Figure S3 | (a) Restoration of trapped cells into the round shape after perfusion is stopped. Scale bar = 20 μm. (b) Cell diameter is measured as the mean of long axis and short axis.

Figure S4 | MCF-7 cells and cytochalasin D treated MCF-7 cells are stained with immunofluorescence. Scale bar = 20 μm.

Figure S5 | Transportability versus cell diameter for small population (N = 947) and large population (N = 4507) MCF-7 cells.

Figure S6 | Atomic force microscopy (AFM) indentation was used to analyze MCF-7 and TPA-induced MCF-7 cells. (a) The bright field image shows indentation of the AFM tip. (b) The number of indicated cell types was plotted versus Young's moduli (kPa). TPA-induced MCF-7 cells have lower average Young's moduli. (c) The AFM image shows MCF-7 cells, scale bar = 20 μm. (d) The AFM image shows TPA-induced MCF-7 cells, scale bar = 20 μm.

Figure S7 | Images of western blotting of E-cadherin, N-cadherin, keratin 18, vinculin, F-actin, snail and GAPDH. All western blot experiments were run under the same experimental conditions. Red lines indicate cropped images shown in Figure 4.

Figure S8 | Cells from the tumor center and periphery were identified on chip. Bright field images show tumor cells stained for human nuclei (green) and counterstained with DAPI (blue), scale bars = 200 μm (a and c, 10× magnification) and 40 μm (b and d, 40× magnification).

Figure S9 | Optimal cutting temperature compound (OCT)-embedded sections from the tumor center and periphery were stained with hematoxylin and eosin, scale bar = 100 μm.

Figure S10 | Tumor tissues were stained with immunofluorescence. Tissues derived from the tumor center (upper panels) and periphery (lower panels) were stained with antibodies against the nuclei, E-cadherin, and vimentin; merged image is on right, scale bar = 50 μm.

Table S1 Proportion of tumor cells dissociated from the tumor center and periphery

	Tumor center	Tumor periphery
Tumor cells (no.)	2203	1531
Total cells (no.)	2270	1610
Tumor cells (%)	97.1	95.1

Movie S1 Separation of cells by size in the DLD structure

Movie S2 Cell capture in the trapping barrier structure

Figure S1

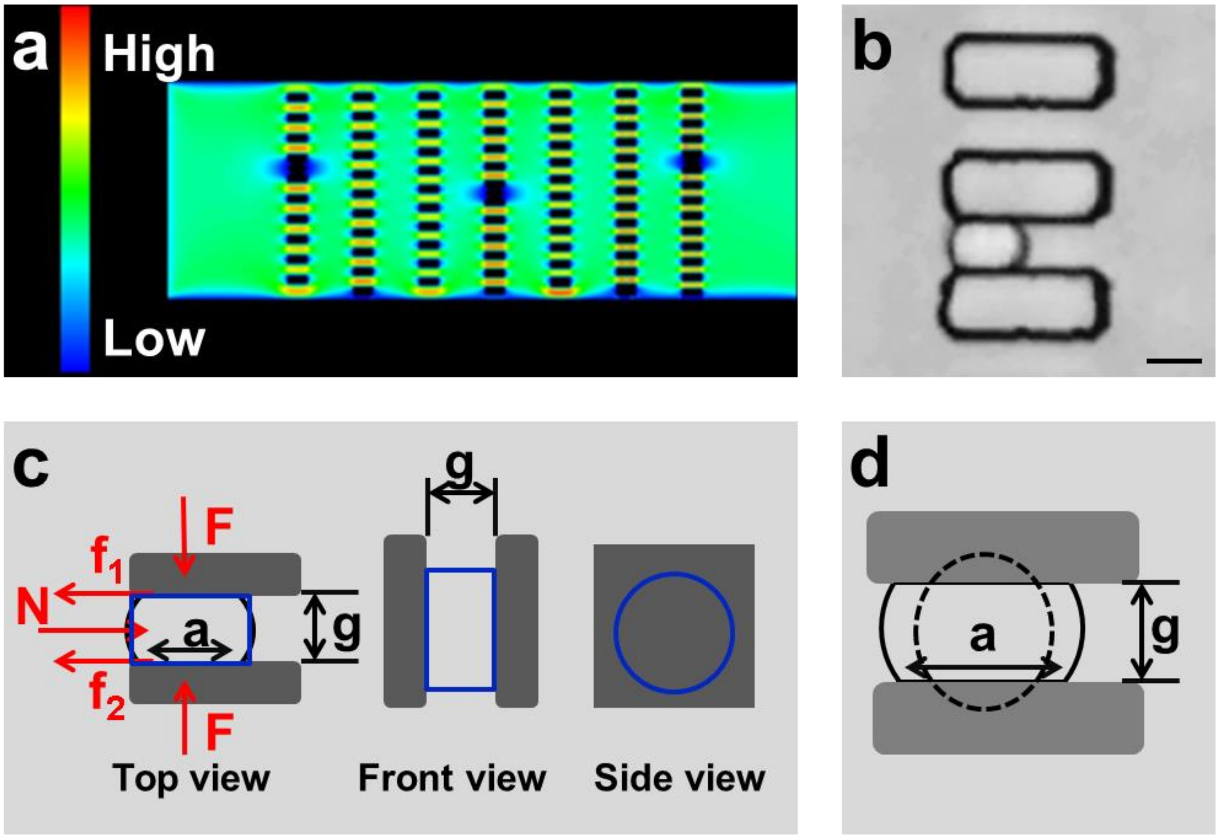

Figure S2

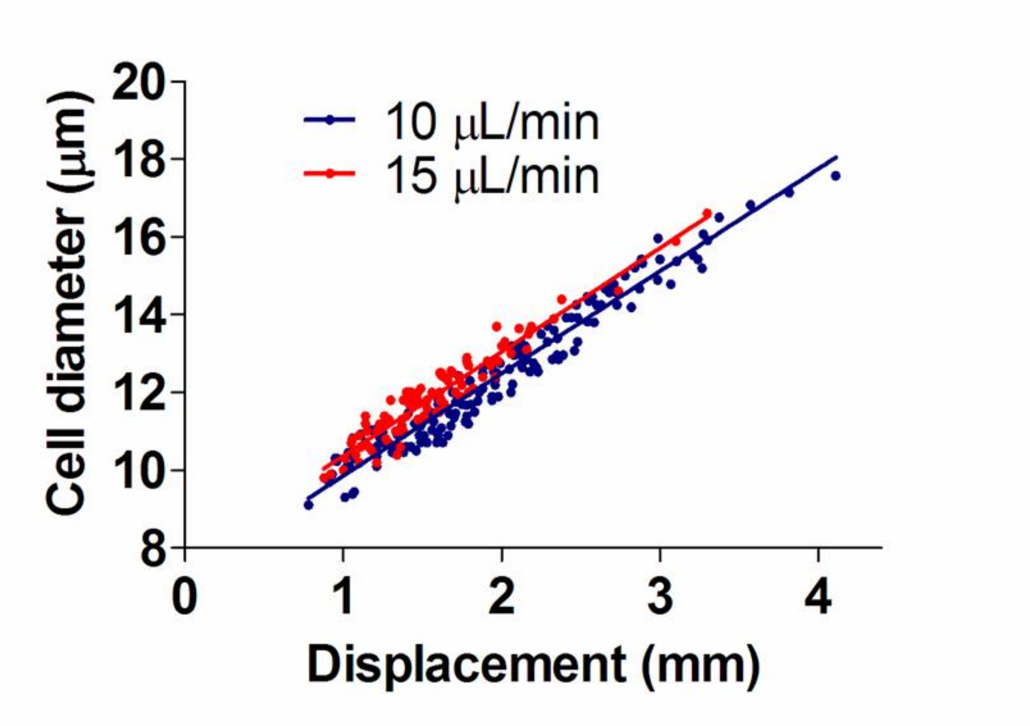

10 μL/min
15 μL/min
Cell diameter (μm)
20
18
16
14
12
10
8
0
1
2
3
4
Displacement (mm)

Figure S3

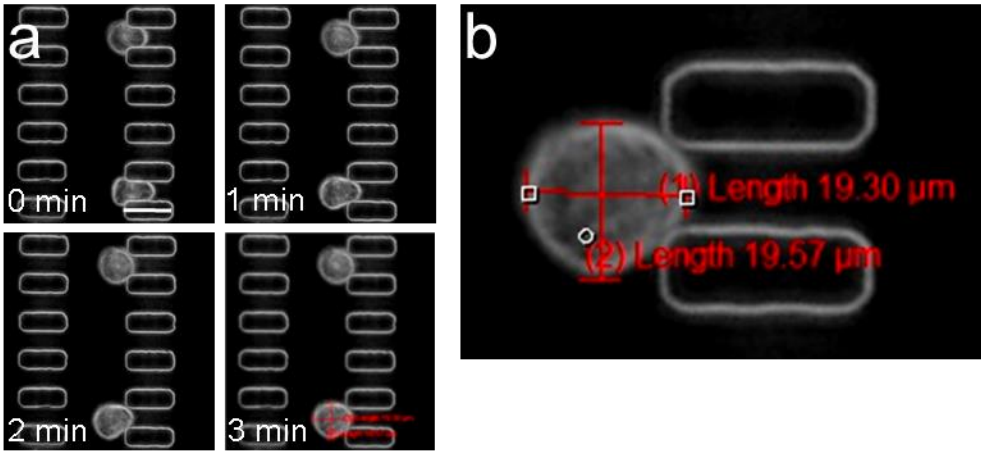

Figure S4

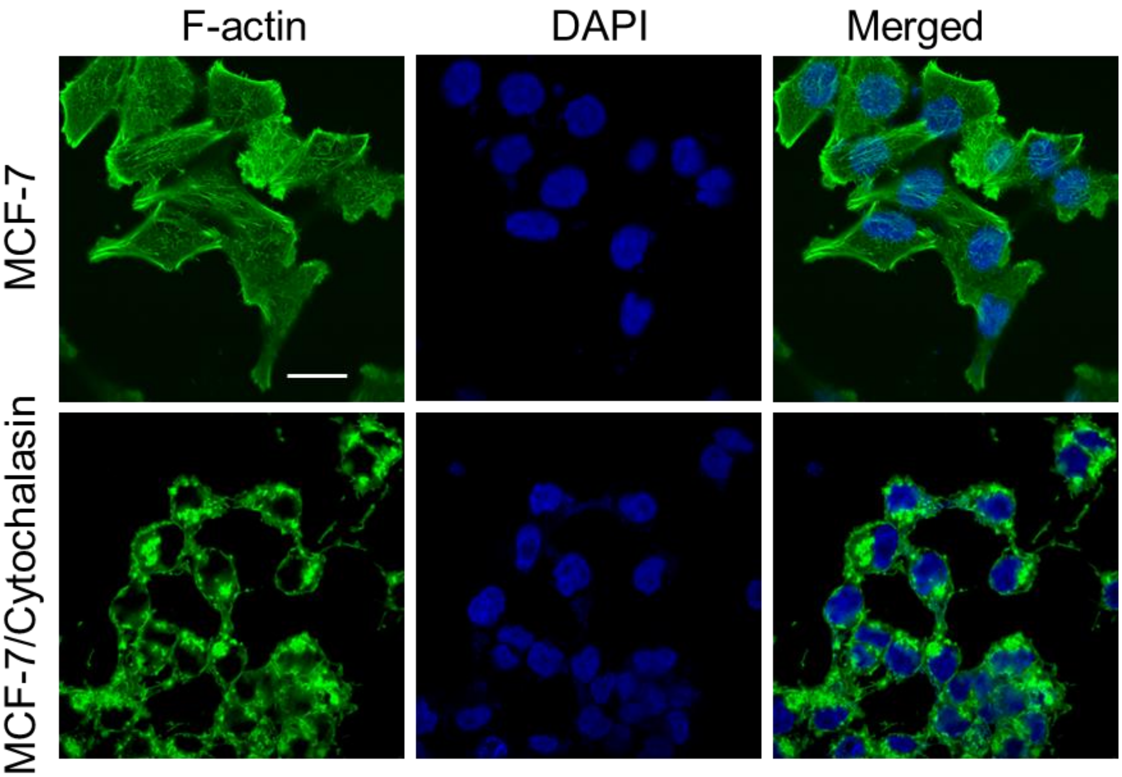

Figure S5

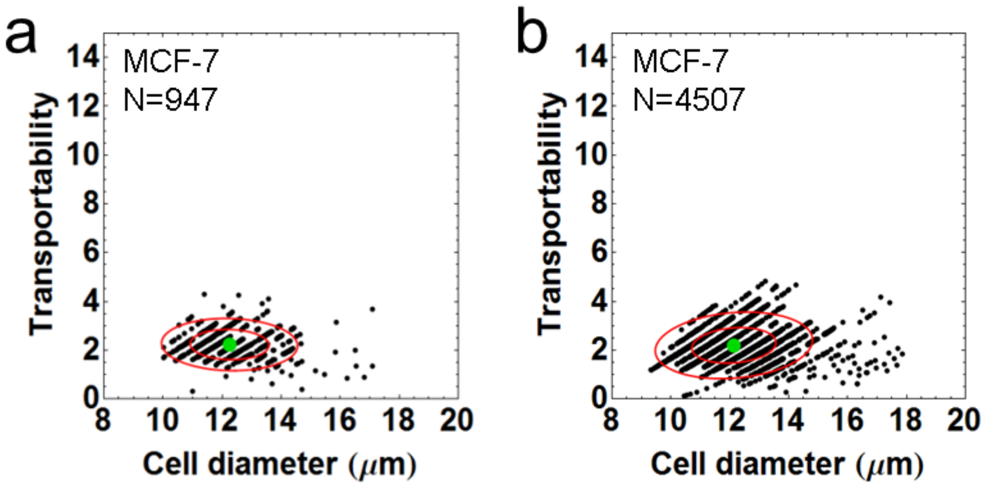
a
MCF-7
N=947
Transportability
Cell diameter (μm)
b
MCF-7
N=4507
Transportability
Cell diameter (μm)

Figure S6

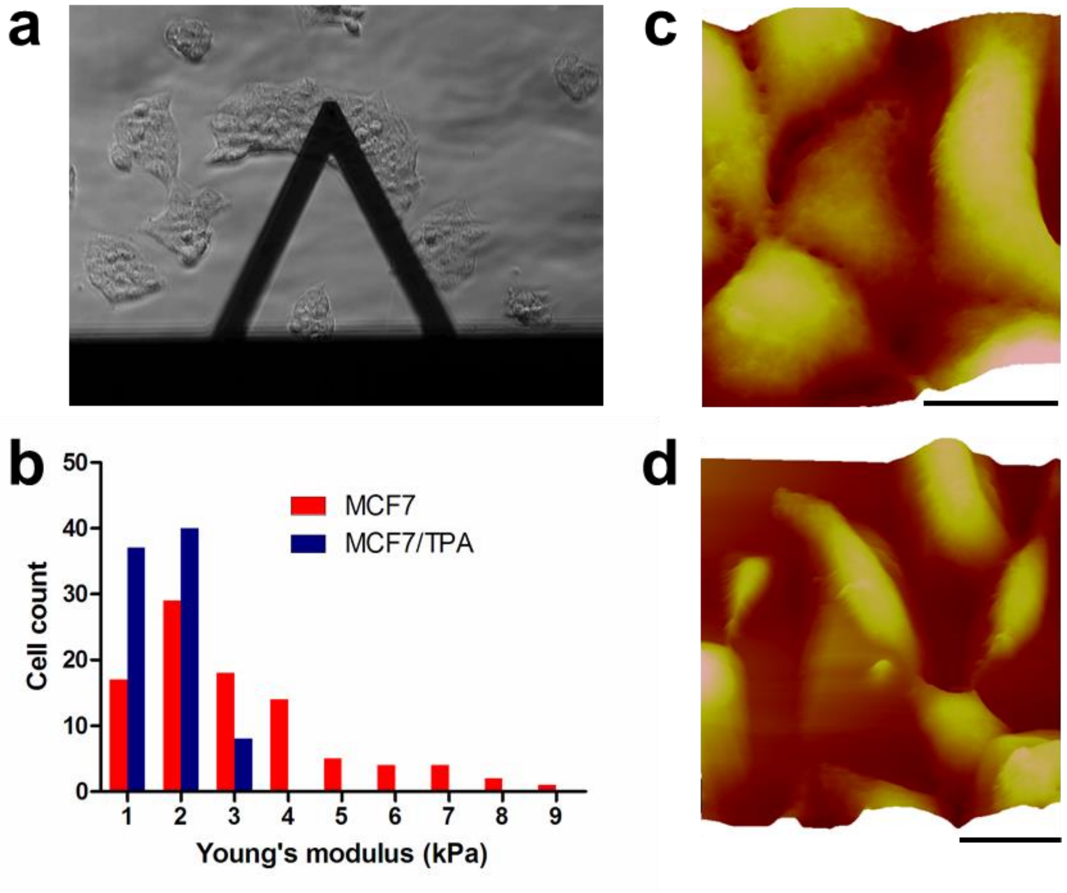
a
c
b
d
MCF7
MCF7/TPA
Cell count
Young's modulus (kPa)
0
10
20
30
40
50
1
2
3
4
5
6
7
8
9

Figure S7

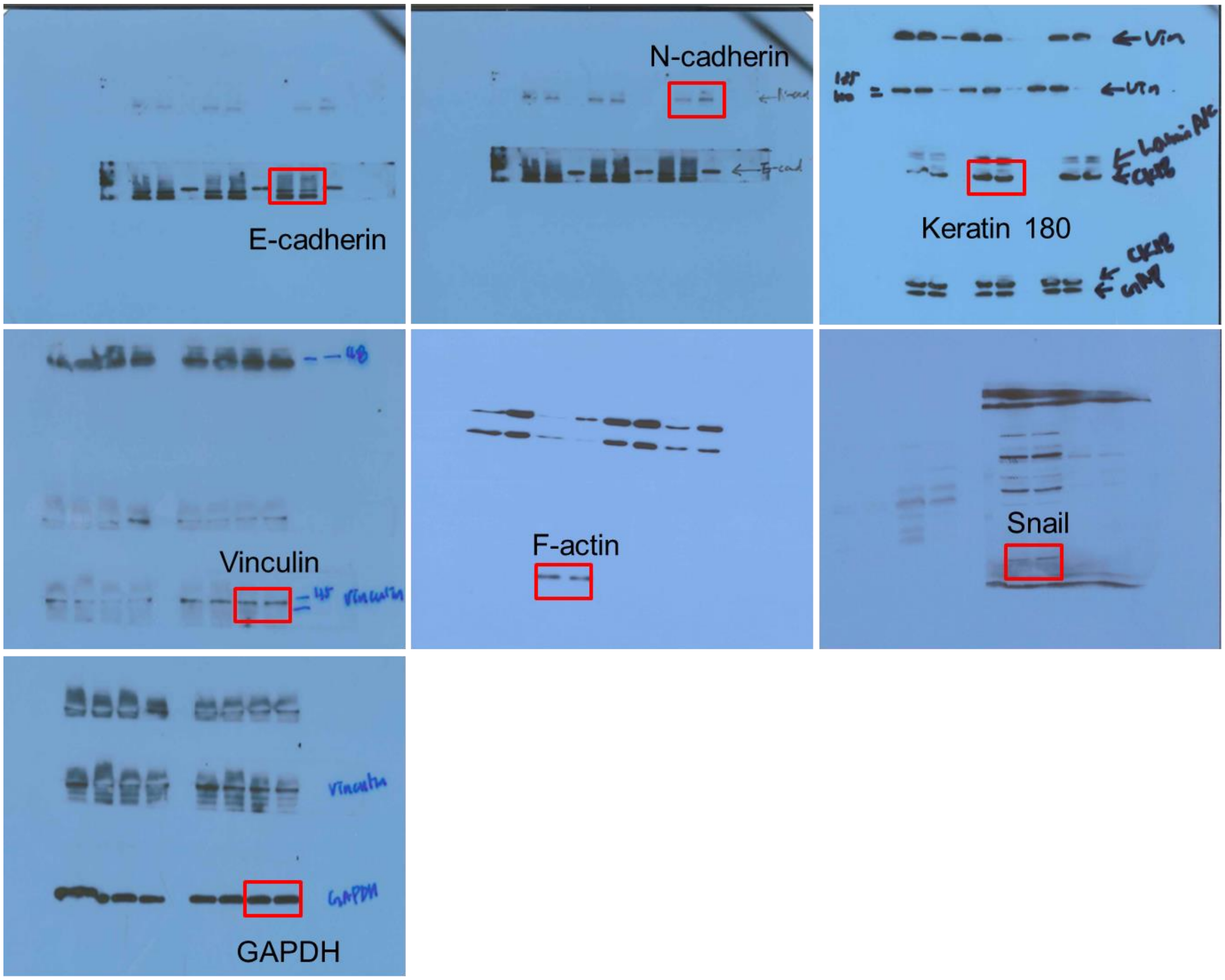
N-cadherin
E-cadherin
Keratin 180
Vinculin
F-actin
Snail
GAPDH

Figure S8

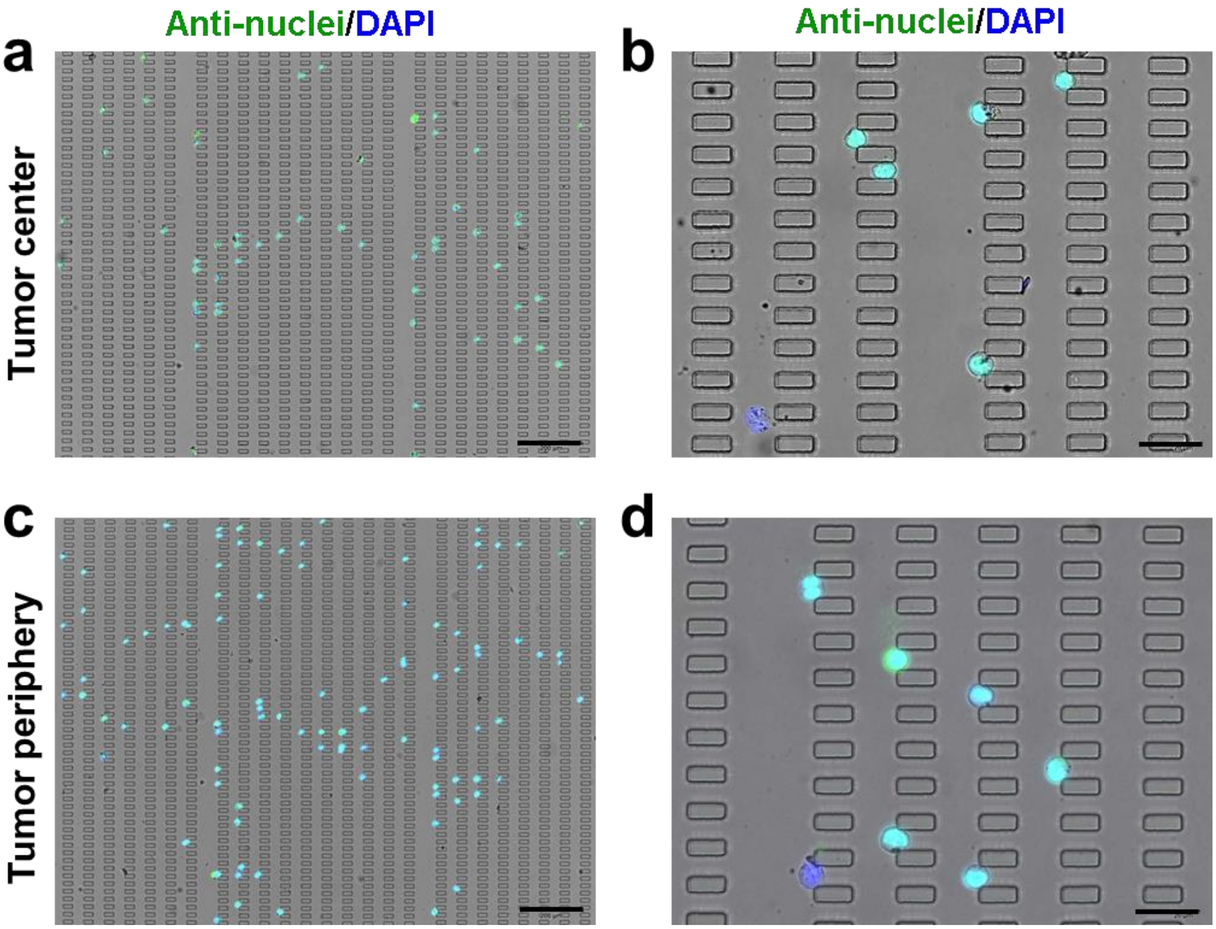

Anti-nuclei/DAPI
Anti-nuclei/DAPI
a
b
Tumor center
c
d
Tumor periphery

Figure S9

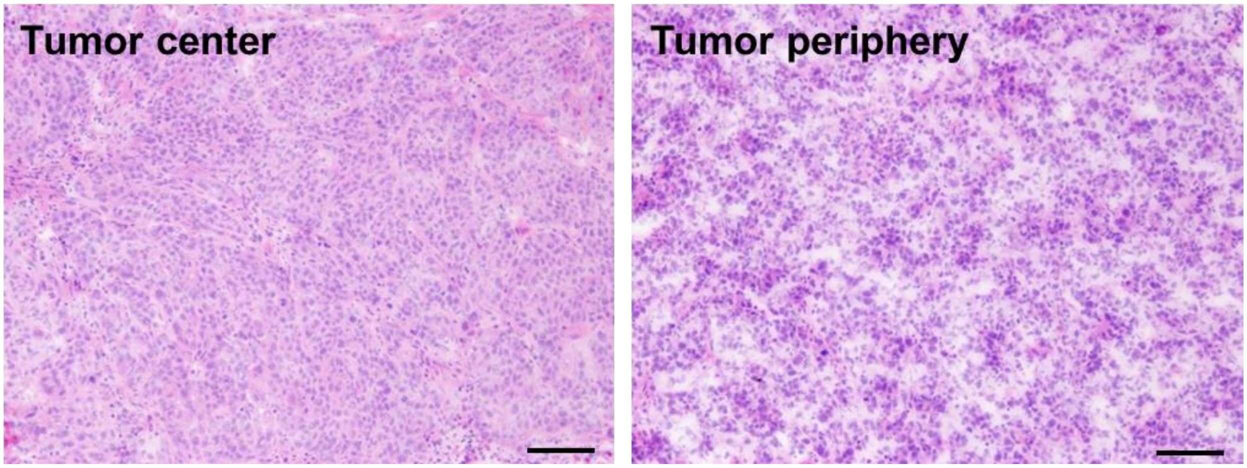

Figure S10

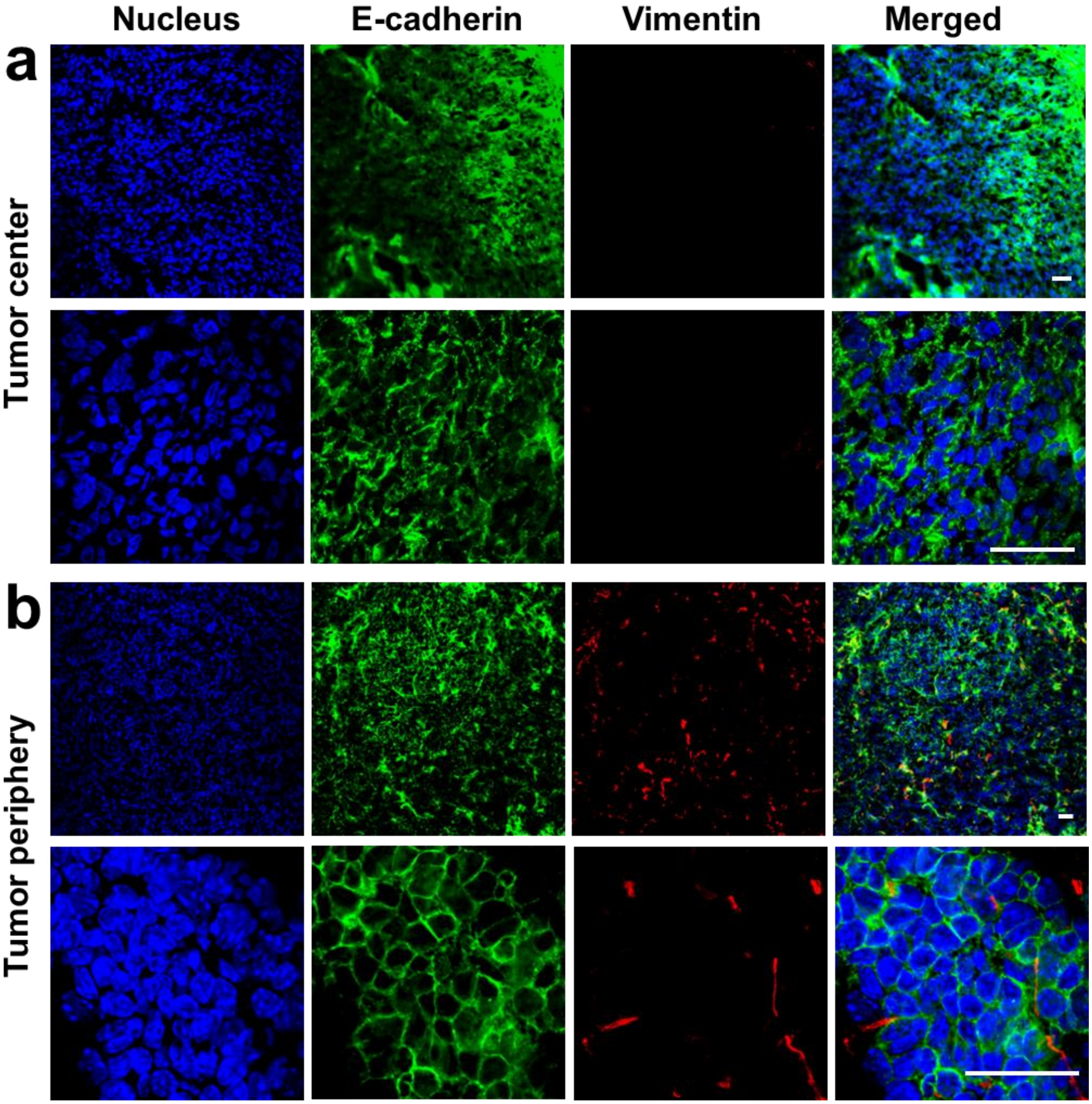
Nucleus
E-cadherin
Vimentin
Merged
a
Tumor center
b
Tumor periphery
